# An external validation study of a practical scoring system for identifying patients at risk of pelvic floor dysfunction following childbirth

**DOI:** 10.1002/ijgo.71099

**Published:** 2026-05-25

**Authors:** Martina Arcieri, Michela Segatti, Filippo Bordin, Doriana Armenise, Maria De Martino, Miriam Isola, Chiara Mattiussi, Elisa Barbui, Sara Filippin, Veronica Tius, Cristina Delneri, Valeria Dialti, Stefano Restaino, Lorenza Driul, Giuseppe Vizzielli

**Affiliations:** ^1^ Clinic of Obstetrics and Gynecology, “Santa Maria Della Misericordia” University Hospital, Azienda Sanitaria Universitaria Friuli Centrale Udine Italy; ^2^ Department of Medicine (DMED) University of Udine Udine Italy; ^3^ Department of Medicine (DMED), Institute of Statistics University of Udine Udine Italy; ^4^ Rehabilitation Medicine Unit Trieste, “San Polo” Hospital ‐ Monfalcone, Azienda Sanitaria Universitaria Giuliano Isontina Monfalcone Italy; ^5^ Physical Medicine and Rehabilitation – Severe Brain Injury Unit / General Rehabilitation, Institute of Physical Medicine and Rehabilitation “Gervasutta,” Azienda Sanitaria Universitaria Friuli Centrale Udine Italy; ^6^ PhD School in Biomedical Sciences, Gender Medicine, Child and Women Health, University of Sassari Sassari Italy

**Keywords:** pelvic floor dysfunction, perineal card, postpartum rehabilitation, urinary incontinence, vaginal delivery

## Abstract

**Objective:**

To prospectively evaluate the perineal card, a risk‐based scoring system, for identifying women at increased risk of postpartum urinary incontinence (UI).

**Methods:**

This prospective observational study included women without previous vaginal delivery who underwent vaginal birth at a tertiary referral center between June 2023 and October 2024. The perineal card was completed at hospital discharge to assess obstetric and perineal risk factors and classify patients into low‐, intermediate‐, and high‐risk groups. Postpartum pelvic floor outcomes were assessed at follow‐up using a standardized screening tool with validated questionnaires. Logistic regression identified factors independently associated with UI, and discriminative performance was evaluated by receiver operating characteristic analysis.

**Results:**

Of 296 enrolled women, 265 were included in the final analysis. At follow‐up, 18.3% reported UI. Women with UI had higher median perineal card scores than asymptomatic women. UI during pregnancy, pre‐pregnancy body mass index (BMI), labor induction, and postpartum urinary retention lasting more than 24 h were independently associated with postpartum UI. The perineal card showed moderate discriminative ability, with an area under the curve of 0.65.

**Conclusion:**

Use of the perineal card at discharge may help identify women at increased risk of postpartum UI and support timely referral for pelvic floor rehabilitation.

## 1 INTRODUCTION

Obstetric pelvic floor dysfunctions (PFDs) include anatomical and functional pelvic floor changes related to pregnancy and childbirth, such as urinary or anal incontinence, prolapse, and sexual dysfunction.^1^ A recent consensus defined obstetric PFDs as anatomical and functional changes of the pelvic floor that arise from pregnancy and childbirth, which may emerge within the first 12 months postpartum.^2^

Although postpartum PFDs are not life‐threatening, these conditions significantly affect quality of life and represent an increasing public health concern.^3^, ^4^

The “perineal card,” developed by the Italian Pelvic Floor Obstetrics Group^5^ (GOIPP) (Table 1), was designed to identify postpartum risk factors and early signs of PFDs, allowing timely referral for pelvic floor rehabilitation. A prospective validation of the perineal card is lacking in the literature.

**TABLE 1:** Perineal card variables in study population.

Perineal card	Score	*N* (%)
**General risk factors**		265
Recurrent bronchitis/cough	1	16 (6.0%)
Heavy physical labor	1	53 (20%)
High‐impact sports/intense physical activity	2	43 (16.2%)
**Urogynecologic risk factors**		
Urinary incontinence in first/s trimester	4	26 (9.8%)
Urinary incontinence in puerperium	4	39 (14.7%)
Severe constipation before pregnancy	1	37 (14%)
Severe constipation during pregnancy	1	83 (31.3%)
Gas or stool incontinence	4	5 (1.9%)
Recurrent urogenital infections	2	26 (9.8%)
**Obstetric risk factors**		
Nulliparous	1	265 (100%)
Weight gain > 14 kg	1	98 (37%)
Breech/abnormal presentation	1	0
Sacral rotation	1	13 (4.9%)
Induced delivery	2	142 (53.6%)
Analgesia	1	192 (72.4%)
Second stage of labor > 60 min	2	149 (56.2%)
Shoulder dystocia maneuvers	2	3 (1.1%)
Vacuum‐assisted delivery	2	54 (20.4%)
Kristeller maneuvers	2	31 (11.7%)
Medio‐lateral episiotomy	2	94 (35.5%)
Midline episiotomy	2	0
First‐degree perineal laceration	1	67 (25.3%)
Second‐degree perineal laceration	2	68 (25.7%)
Third‐degree perineal laceration	4	9 (3.4%)
Fourth‐degree perineal laceration	4	1 (0.4%)
Perineal hematoma	1	1 (0.4%)
Perineal infection	1	1 (0.4%)
Catheterization	1	126 (47.5%)
Manual removal of a retained placenta	2	6 (2.3%)
Neonatal head circumference > 35	1	53 (20.0%)
Perineal card score (median [range])		10 (2–25)

Pelvic floor muscle training (PFMT) is recommended as the first‐line treatment for urinary incontinence and may also benefit other pelvic floor disorders. Evidence suggests that targeted postpartum PFMT may be more effective than universal interventions.^6^, ^7^, ^8^, ^9^

This study prospectively evaluated the perineal card in primiparous women, assessing its accuracy in identifying individuals at risk of PFDs to facilitate referral for early PFMT.

## 2 MATERIALS AND METHODS

This manuscript presents a prospective observational study including primiparous women undergoing vaginal delivery at a tertiary center. The study was approved by the Institutional Review Board (protocol ID: 74/2023) and conducted at the Clinic of Gynecology and Obstetrics of “Santa Maria della Misericordia” University Hospital and the Institute of Physical Medicine and Rehabilitation “Gervasutta” in Udine, Italy, from June 2023 to October 2024. Women were enrolled at the preadmission appointment before delivery. All patients signed an informed consent form before participating in the study.

Inclusion criteria included women after vaginal delivery and without a prior vaginal delivery, single full‐term pregnancies (from 37^+0^ to 42^+0^ gestational weeks), both induced and non‐induced pregnancies, and first vaginal deliveries following a cesarean section. Exclusion criteria comprised multiparous women, twin or multiple pregnancies, maternal neurologic pathologies, previous perineal surgeries, collagen diseases, the presence of PFDs prior to pregnancy, body mass index (BMI, calculated as weight in kilograms divided by the square of height in meters) greater than 30 or less than 18 (pre‐pregnancy), newborn weights exceeding 4000 g or below 2000 g at birth, and births occurring before the 37th gestational week, cesarean sections.

Medical history, general, and obstetric risk factors for developing PFDs, along with data from perineal evaluations, were organized and stored in an anonymous, structured database. Patients' medical records were consulted, and an interview was conducted to fill out the perineal card (Table 1).

The perineal card provides complementary items for assessing the risk of onset of perineal dysfunctions. It aims to divide women into three risk groups for developing PFDs in the postpartum period based on their score: a score of ≤ 10 indicates the low‐risk group, which entails admission to re‐education and behavioral therapy sessions only; a score of 11–25 indicates the intermediate‐risk group, which entails admission to short‐cycle education/rehabilitation sessions; and a score > 25 indicates the high‐risk group, which entails referral to the urogynecology/proctology outpatient clinic and subsequent rehabilitation.

For each woman, the perineal card (Table 1) and Italian Society of Urodynamics' (SIUD) delivery and pelvic dysfunctions card^10^ (Figure S1) were completed at the time of discharge (T0) following delivery. During follow‐up, women were evaluated using the SIUD postpartum screening card^10^ (Figure S2). The form gathers data on urinary and anal incontinence, prolapse, pain, dyspareunia, and dysfunction of the perineal muscles, using internationally validated tools.

For patients identified as being at high risk of perineal dysfunction—specifically, those exhibiting symptoms of urinary and/or fecal incontinence upon discharge and/or experiencing vagino‐perineal laceration of III–IV degree—the SIUD postpartum screening card was duly completed within three months following delivery (T1). Furthermore, in accordance with our internal protocol designated for patients classified as high risk for perineal dysfunction, these individuals subsequently underwent a psychiatric evaluation at the Institute of Physical Medicine and Rehabilitation “Gervasutta.” They engaged in a 10‐session individualized perineal rehabilitation program, during which a variety of techniques were utilized (including kinesiotherapy, electrostimulation, and analgesic electrotherapy) tailored to the specific issues faced by each patient.^8^, ^9^ Women who did not present symptoms of PFD at the time of discharge (T0) were evaluated subsequently (6–18 months post‐delivery) to ascertain the correlation between the perineal card score and the presence or absence of uro‐gynecologic dysfunction, as assessed via the completion of the SIUD postpartum screening card (T1). The follow‐up protocol is illustrated in Figure 1.

**FIGURE 1:**
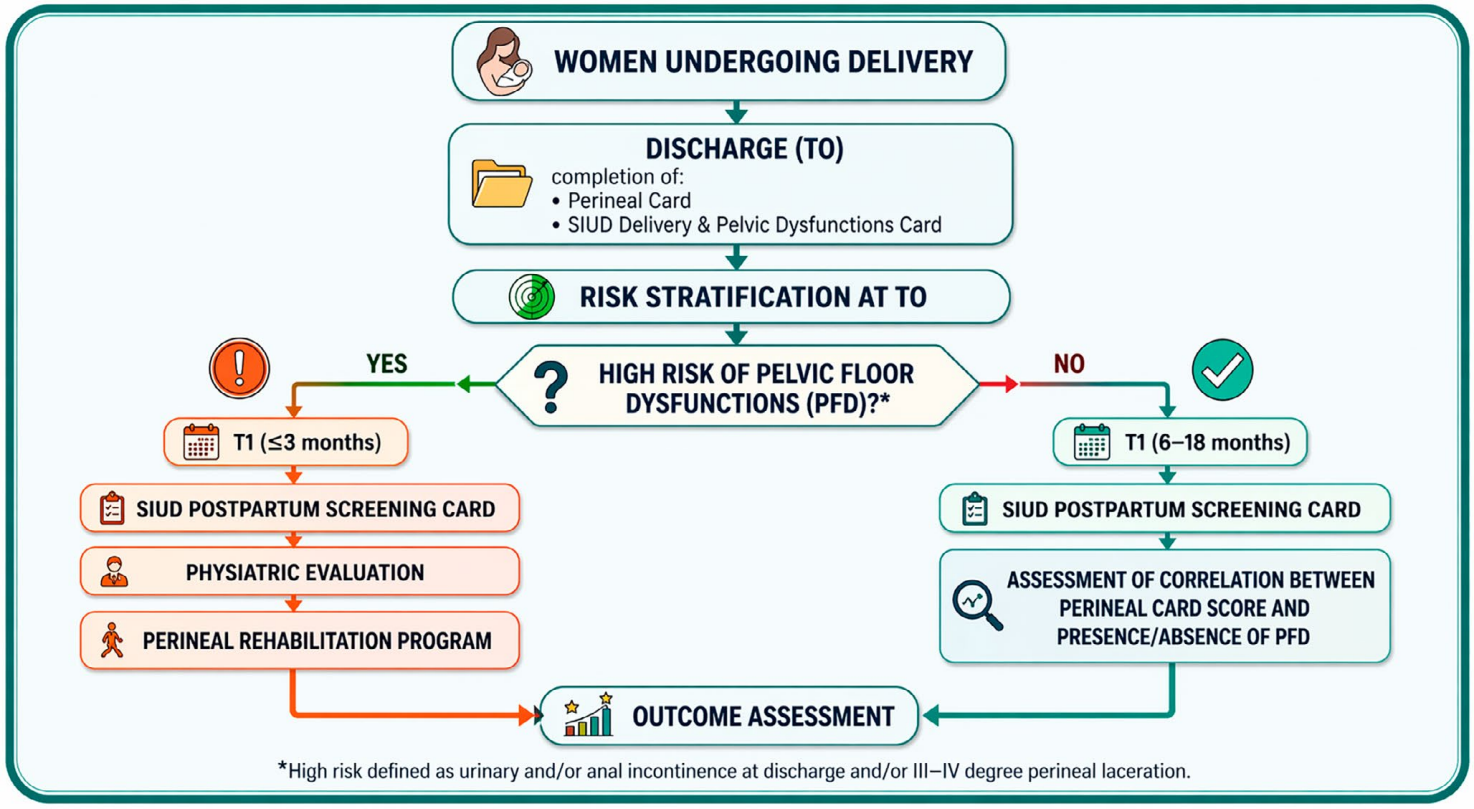
Follow‐up protocol flow chart.

### 2.1 Statistical analysis and sample size

The sample size was calculated assuming a 20% prevalence of postpartum urinary incontinence (UI), resulting in a required minimum of 295 primiparous women, including a 20% allowance for follow‐up dropout. Descriptive statistics were used to summarize quantitative and qualitative variables. Group comparisons were performed using Chi‐square or Fisher exact tests for categorical variables, Student's *t*‐test for normally distributed data, and the Mann–Whitney *U* test for non‐normal distributions. Univariable and multivariable logistic regression analyses were conducted to identify factors associated with UI, reporting odds ratios (ORs) with 95% confidence intervals. Receiver operating characteristic (ROC) analysis was used to assess the ability of the perineal score to discriminate between women with and without UI. Statistical significance was set at a *P* value less than 0.05, and analyses were performed using RStudio (version 2026.01.1 + 403).

## 3 RESULTS

A total of 296 women were enrolled, of which 31 (10.5%) were excluded (Figure 2). Consequently, 265 women were included in the final analysis, with a mean age of 31.8 years (19–47 years). The mean gestational age at delivery was 279 days (259–292 days), equal to 39 weeks and 6 days.

**FIGURE 2:**
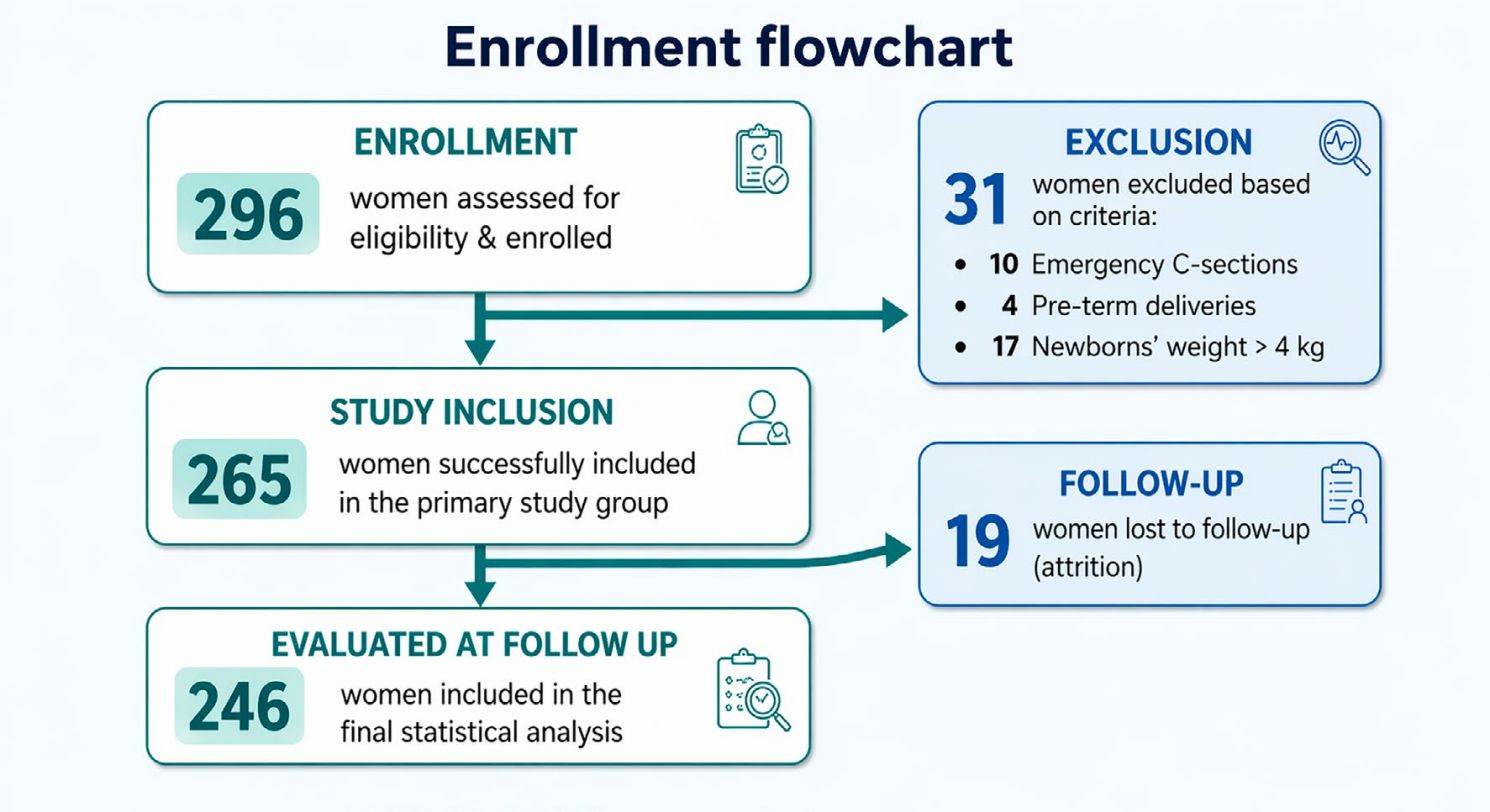
Enrollment flow chart.

Table S1 presents the perineal card variables for the study population. A total of 16 patients had a prior cesarean section. The median gestational weight gain was 13 kg (1–35 kg). The mean BMI at the time of delivery was 26.7 (19.4–38), while the mean pre‐pregnancy BMI was 21.9 (16.5–30). During the first and second trimesters of pregnancy, 26 women (9.81%) reported experiencing UI. However, when the third trimester is included in the analysis, this figure significantly increases to 95 women (35.8%). Among these 95 women, 87 experienced stress incontinence, while eight (3.02%) reported urgency incontinence.

The mean duration of the second stage of labor was 84. 5 ± 55.1 min. Precipitous labor or labor induction occurred in 139 women (52.4%). Various induction methods were employed based on clinical indications: 33 women (12.4%) received oxytocin infusion, 27 (10.2%) were induced with oral misoprostol followed by oxytocin infusion, 23 (8.7%) with oral misoprostol alone, 11 (4.1%) with amniotomy and oxytocin, eight (3%) with Cook balloon placement and oxytocin, and six (2.3%) with a combination of oral misoprostol, Cook balloon placement, amniotomy, and oxytocin. The perineum remained intact in 28 women (10.6%), whereas 67 (25.3%) experienced first‐degree perineal tears, 68 (25.7%) had second‐degree tears, and nine (3.4%) sustained third‐degree tears—specifically, three cases were classified as grade B and six as grade A. One case (0.4%) involved a fourth‐degree tear. The mean birth weight was recorded at 3294.3 ± 376 g while the mean head circumference was 34.1 ± 1.3 cm. Upon analyzing the positions in which childbirth occurred, it was observed that 105 women (39.6%) delivered in the gynecologic position, 69 (26%) in the supine position, 41 (15.5%) in the lateral position, 19 (7.2%) on all fours, 18 (6.8%) in a semi‐sitting position, four (1.5%) standing, four (1.5%) squatting, three (1.1%) utilized a rope, and two (0.7%) employed the Dutch position stool.

The median perineal card score of 265 enrolled women was found to be 10, with a range from 2 to 25 points. Specifically, 143 (54%) women achieved a score of less than 11 points, 121 (45.7%) scored between 11 and 25, and one (0.3%) scored greater than 25 points (Figure 3).

**FIGURE 3:**
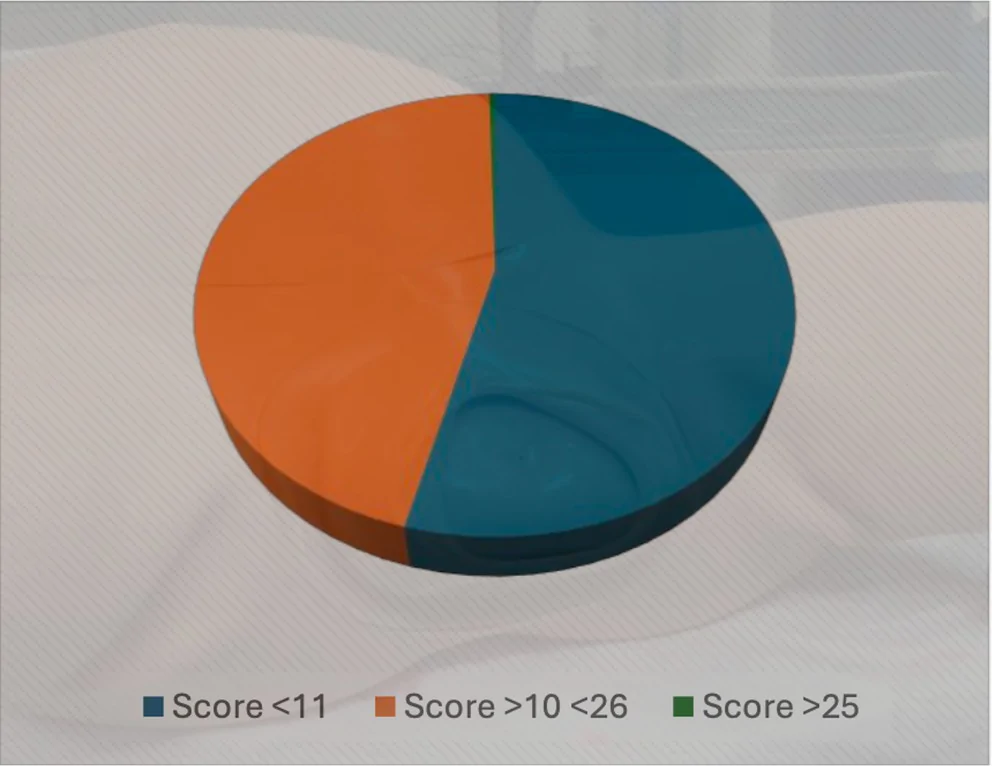
Distribution of perineal card scores.

Among the 265 women, 246 were evaluated following delivery, with a mean follow‐up period of 277 days (range: 33–462 days, SD 76.3). A total of 21 patients (8.5%) were assessed within the first 6 months post‐delivery due to a high risk of perineal dysfunction. A total of 19 women (7.2%) were lost to follow‐up. Table 2 provides a summary of the characteristics of the women assessed. A total of 45 (18.3%) women reported experiencing UI, and eight (3%) reported anal incontinence during their follow‐up visit. Table 3 presents the association between the variables investigated at discharge and the presence of UI. A univariable analysis indicated a statistically significant association between the presence of UI at follow‐up and UI during pregnancy, induced labor, epidural analgesia, pre‐pregnancy BMI and postpartum urinary retention lasting more than 24 h. However, the association with epidural analgesia did not remain significant in the multivariable analysis (Table 4). The median perineal score was 9 in women without UI at follow‐up, compared to 11 in those with UI (*P* = 0.001) (Figures 4 and 5).

**TABLE 2:** Characteristics of women at follow‐up.

Patients	*N* 246 (%)
Patients evaluated by clinical evaluation	223 (90.7%)
Patients evaluated by phone interview	23 (9.3%)
Vagino‐perineal lacerations	
Intact perineums	96 (39%)
First‐degree tears	72 (29%)
Second‐degree tears	69 (28%)
Third‐degree tears	8 (3.3%)
Fourth‐degree tears	1 (0.4%)
Median perineal score (IQR)	10.0 (7.0, 13.0)
Urinary incontinence	
No incontinence	201 (81.7%)
Urge incontinence	39 (15.9%)
Stress incontinence	4 (1.6%)
Mixed	2 (0.8%)
Anal incontinence	
No incontinence	238 (96.8%)
Only gas incontinence	6 (2.4%)
Only fecal incontinence	0
Mixed	2 (0.8%)
Resumption of sexual intercourse	
Yes	214 (87.0%)
No	34 (13.0%)
Median weeks (IQR)	9 (1–34)
Mean IC‐QSF score (SD)	1.6 (3.8)
Mean Wexner score (SD)	0.8 (0.5)
Visual analog scale perineal pain Mean (SD)	0.1 (0.7)
Visual analog scale dyspareunia Mean (SD)	1.0 (2.2)
Mean Marinoff score (SD)	0.3 (0.6)
Visual analog scale reduced sensibility during sexual intercourse Mean (SD)	0.6 (1.7)
Anterior POP	
No	196 (79.7%)
Stage I	25 (10.2%)
Stage II	2 (0.8%)
Stage III	0
Central POP	
No	195 (79.3%)
Stage I	27 (11.0%)
Stage II	1 (0.4%)
Stage III	0
Posterior POP	
No	220 (89.4%)
Stage I	3 (1.2%)
Stage II	0
Stage III	0
PC test Oxford scale	
Median (IQR) right	3 (2–4)
Median (IQR) left	3 (2–3.5)
Median (IQR) endurance	1 (0–2)
Pregnant during follow‐up	6 (2.4%)

Abbreviations: IQR, interquartile range; PC, pubococcygeus; POP, pelvic organ prolapse; SD, standard deviation.

**TABLE 3:** Descriptive variables by UI.

Characteristic	No UI *N* = 201^a^	Yes UI *N* = 45^a^	*p* ^b^
Age	31 (28–35)	32 (29–36)	0.503
Recurrent bronchitis/cough	10 (5.0%)	4 (8.9%)	0.562
Heavy physical labor	42 (21%)	7 (16%)	0.742
Intense physical activity	36 (18%)	5 (11%)	0.562
UI in first/s trimester	16 (8.0%)	9 (20%)	0.151
UI in puerperium	22 (11%)	12 (27%)	0.069
Constipation	71 (35%)	12 (27%)	0.562
Gas incontinence during pregnancy	1 (0.5%)	0 (0%)	1.000
Fecal incontinence in puerperium	3 (1.5%)	1 (2.2%)	0.824
Recurrent urogenital infections	18 (9.0%)	7 (16%)	0.503
Weight gain (kg)	12.0 (10.0–15.0)	13.5 (10.0–17.0)	0.824
Weight gain > 14 kg	68 (34%)	18 (40%)	0.742
Sacral rotation	10 (5.0%)	2 (4.4%)	1.000
Induced labor	**95 (47%)**	**33 (73%)**	**0.028**
Analgesia	137 (68%)	38 (84%)	0.151
Duration of second stage (min)	71 (38–120)	87 (43–130)	0.773
Second stage of labor > 60 min	109 (54%)	26 (58%)	0.856
Shoulder dystocia maneuvers	2 (1.0%)	0 (0%)	1.000
Vacuum‐assisted delivery	39 (19%)	9 (20%)	1.000
Kristeller maneuver	20 (10.0%)	6 (13%)	0.824
Episiotomy	66 (33%)	19 (42%)	0.555
Intact perineum	76 (38%)	20 (44%)	0.562
Perineal laceration – grade 1	62 (31%)	10 (22%)	
Perineal laceration – grade 2	56 (28%)	13 (29%)	
Perineal laceration – grade 3	7 (3.5%)	1 (2.2%)	
Perineal laceration – grade 4	0 (0%)	1 (2.2%)	
Perineal hematoma	0 (0%)	1 (2.2%)	0.503
Catheterization	90 (45%)	26 (58%)	0.457
Manual removal of a retained placenta	5 (2.5%)	1 (2.2%)	1.000
Neonatal head circumference (cm)	34.10 (33.00–35.00)	34.00 (33.50–35.00)	0.824
Neonatal head circumference > 35	41 (21%)	9 (20%)	1.000
Neonatal weight (g)	3304 (3030–3544)	3332 (3081–3640)	0.745
Delivery position – type 1	83 (41%)	15 (33%)	0.895
Delivery position – type 2	50 (25%)	11 (24%)	
Delivery position – type 3	19 (9.5%)	4 (8.9%)	
Delivery position – type 4	31 (15%)	8 (18%)	
Delivery position – type 5	12 (6.0%)	5 (11%)	
Delivery position – type 6	6 (3.0%)	2 (4.4%)	
Pre‐pregnancy BMI	21.3 (19.9–23.2)	22.0 (20.6–24.8)	0.168
BMI at delivery	26.0 (24.2–28.3)	26.6 (24.9–30.1)	0.503
Postpartum urinary retention > 24 h	6 (3.0%)	6 (13%)	0.084
UI during pregnancy	59 (29%)	22 (49%)	0.084
Dyspareunia during pregnancy	29 (14%)	10 (22%)	0.503
Perineal score	**9.0 (6.0–12.0)**	**11.0 (10.0–15.0)**	**0.028**

*Note*: BMI, calculated as weight in kilograms divided by the square of height in meters. Type delivery position: (1) Gynecologic/lithotomy, (2) lateral, (3) all‐fours (hands and knees), (4) semi‐seated, (5) squatting/standing, (6) rope/stool. Bold values indicate *p*‐value < 0.050.

Abbreviations: BMI, body mass index; IQR, interquartile range; UI, urinary incontinence.

^a^ *N* (%); median (IQR).

^b^ Fisher exact test; Pearson's chi‐squared test; Mann Whitney *U* test.

**TABLE 4:** Univariable and multivariable logistic regression for risk of UI.

Risk factor	Univariable analysis			Multivariable analysis		
	OR	95% CI	*p*	OR	95% CI	*p*
Age	1.06	0.99, 1.13	0.089			
Recurrent bronchitis/cough	1.86	0.49, 5.88	0.312			
Heavy physical labor	0.70	0.27, 1.59	0.419			
Intense physical activity	0.57	0.19, 1.44	0.274			
UI in first/s trimester	2.89	1.15, 6.94	**0.020**			
UI in puerperium	2.96	1.31, 6.49	**0.008**			
Constipation	0.67	0.31, 1.34	0.269			
Fecal incontinence in puerperium	1.50	0.07, 12.03	0.728			
Recurrent urogenital infections	1.87	0.69, 4.63	0.191			
Weight gain (kg)	1.01	0.94, 1.08	0.805			
Weight gain > 14 kg	1.30	0.66, 2.52	0.434			
Sacral rotation	0.89	0.13, 3.53	0.881			
Precipitous/induced labor	3.07	1.53, 6.50	**0.002**	2.51	1.19, 5.55	**0.018**
Analgesia	2.54	1.13, 6.48	**0.034**	2.31	0.97, 6.19	0.074
Duration of second stage (min)	1.00	0.99, 1.01	0.489			
Second stage of labor > 60 min	1.15	0.60, 2.24	0.666			
Vacuum‐assisted delivery	1.04	0.44, 2.26	0.927			
Kristeller maneuver	1.39	0.48, 3.52	0.506			
Episiotomy	1.49	0.76, 2.88	0.233			
Intact perineum	1					
Perineal laceration – grade 1	0.61	0.26, 1.38	0.248			
Perineal laceration – grade 2	0.88	0.40, 1.91	0.752			
Perineal laceration – grade 3	0.54	0.03, 3.31	0.578			
Catheterization	1.69	0.88, 3.28	0.117			
Manual removal of placenta	0.89	0.05, 5.71	0.917			
Neonatal head circumference (cm)	1.07	0.84, 1.37	0.574			
Head circumference > 35 cm	0.97	0.41, 2.10	0.940			
Neonatal weight (g)	1.00	0.99, 1.01	0.545			
Delivery position – type 1	1					
Delivery position – type 2	1.22	0.51, 2.84	0.652			
Delivery position – type 3	1.16	0.31, 3.65	0.805			
Delivery position – type 4	1.43	0.53, 3.63	0.463			
Delivery position – type 5	2.31	0.66, 7.26	0.165			
Delivery position – type 6	1.84	0.25, 8.92	0.478			
Pre‐pregnancy BMI	1.14	1.03, 1.26	**0.014**	4.04	1.02, 1.28	**0.022**
BMI at delivery	1.09	0.99, 1.20	0.060			
Postpartum urinary retention > 24 h	5.00	1.49, 16.78	**0.008**	4.04	1.02, 1.28	**0.022**
UI during pregnancy	2.30	1.19, 4.46	**0.013**	2.55	1.25, 5.24	**0.010**
Dyspareunia during pregnancy	1.69	0.73, 3.70	0.199			

*Note*: BMI, calculated as weight in kilograms divided by the square of height in meters. Type delivery position: (1) Gynecologic/lithotomy, (2) lateral, (3) all‐fours (hands and knees), (4) semi‐seated, (5) squatting/standing, (6) rope/stool. Bold values indicate *p*‐value < 0.050.

Abbreviations: BMI, body mass index; CI, confidence interval; OR, odds ratio; UI, urinary incontinence.

**FIGURE 4:**
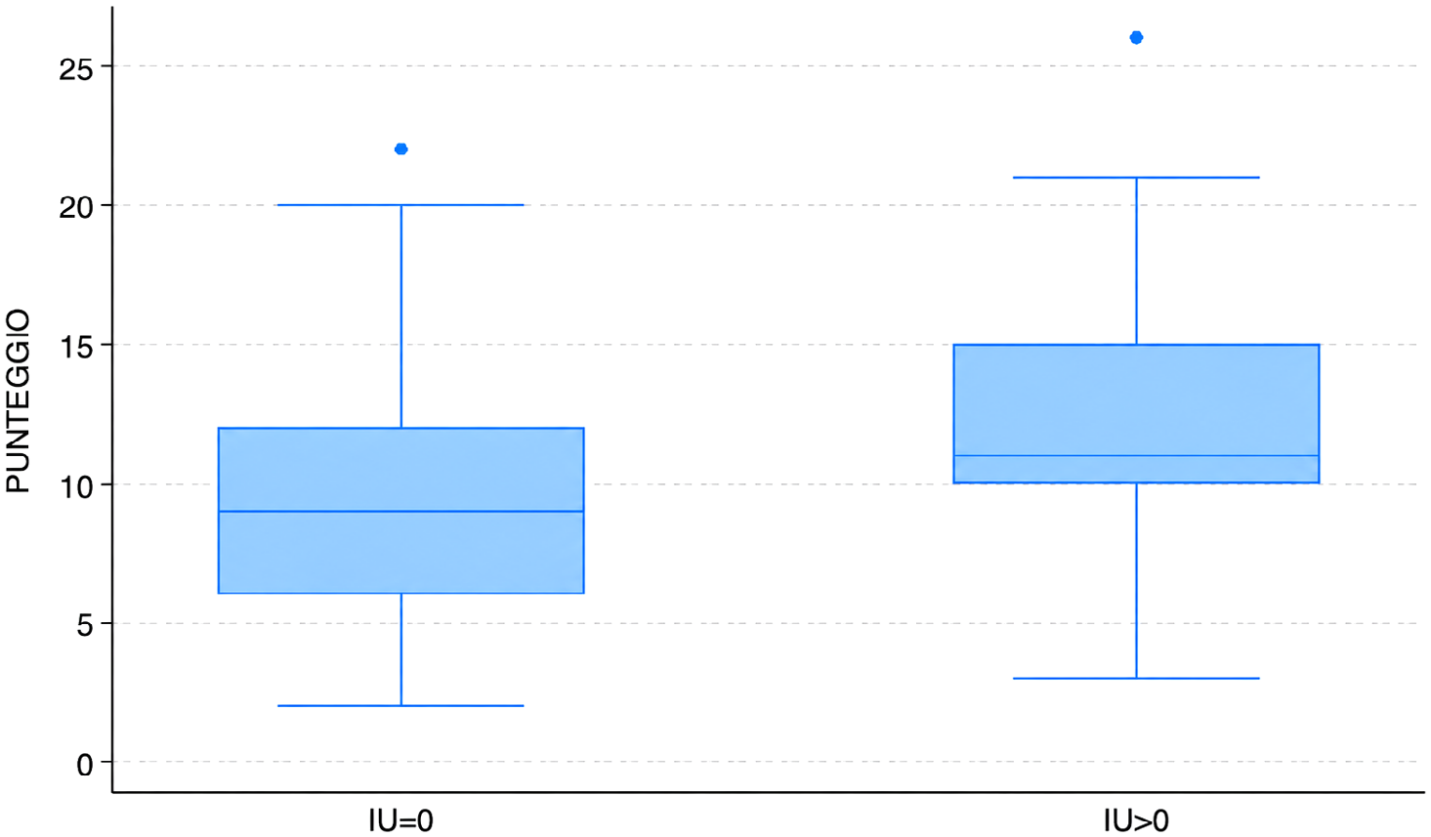
Box plot. UI, urinary incontinence.

**FIGURE 5:**
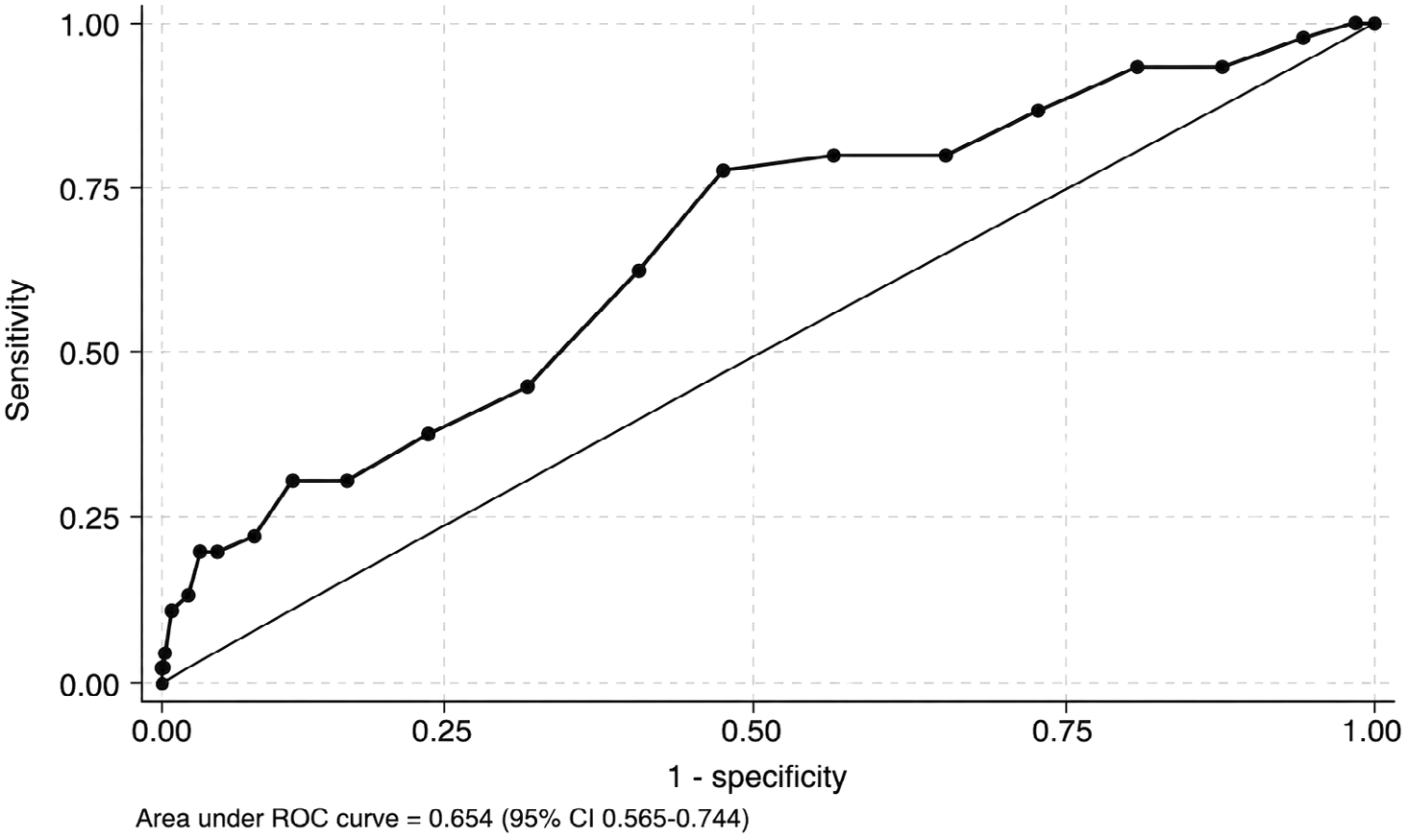
Receiver operating characteristic (ROC) curve for the score in detecting urinary incontinence (UI).

## 4 DISCUSSION

This study is the first prospective investigation validating the perineal card as a clinical tool for identifying women at risk of postpartum UI and other PFDs. So far, the perineal card has not been validated by scientific studies, limiting its adoption across Italian healthcare institutions.

Obstetric PFDs impact women's quality of life, making prevention and early treatment crucial. Restoring the pelvic floor's musculoaponeurotic function first involves validated conservative approaches like PFMT. Currently, there are no consistent methods to identify postpartum women at higher risk for PFDs. PFMT may be offered as preventive care to all pregnant and postpartum women or targeted at symptomatic individuals.

To this end, the perineal card was developed. It adopts a risk‐based approach, aiming to identify women predisposed to PFDs to hospital discharge. This standardized tool assists in harmonizing clinical practice and comprises a checklist with a final score, indicating which women should be referred for PFMT.

Our study demonstrated that the perineal card showed moderate discriminatory power for UI (AUC = 0.65). Women with higher scores had a significantly greater incidence of UI at follow‐up, enabling early risk stratification and timely referral to PFMT.

Although several studies have developed checklists to identify women at risk of postpartum PFDs, the condition's multifactorial nature and numerous influencing factors remain a major challenge. A study conducted in 2014^11^ introduced a scoring system referred to by the acronym UR‐CHOICE. This scoring system aims to predict the future risk of developing PFDs; however, external validation studies are currently lacking. Soligo et al. identified four factors as risk indicators for PFDs: symptoms prior to pregnancy, symptoms during pregnancy, the utilization of a vacuum extractor, and severe perineal tears; showing high combined sensitivity (82%) but low specificity (39%).^12^

Our study suggests that the risk factors associated with the presence of UI during the follow‐up visit may include UI during pregnancy, pre‐pregnancy BM, precipitous or induced labor, epidural analgesia, and postpartum urinary retention lasting more than 24 h. In our research, UI during pregnancy was confirmed as a risk factor, particularly considering that our exclusion criteria encompassed symptoms prior to pregnancy. A total of 95 women (35.8%) experienced UI during pregnancy, with 26 of these cases (27.3%) occurring in the first or second trimester. Among the 69 women who developed gestational UI in the third trimester, 20.3% also reported UI in the postpartum period. Similarly, 23% of the 26 women who experienced gestational UI during the first or second trimester reported UI in the postpartum period. Similar data were reported in a 2021 meta‐analysis,^13^ where the prevalence of UI was noted to be about 28% during the first and second trimesters and rising to about 34% in the third trimester.

As our results also demonstrate, stress incontinence is the most common type of UI during pregnancy, with most affected women experiencing small urine leaks. The incidence of UI increases as pregnancy progresses, and its high prevalence is attributable to various factors, including changes in collagen and hormonal fluctuations, physiological weight gain, and increased intra‐abdominal pressure on the bladder and perineum.^14^

Evidence on the impact of labor induction on pelvic floor outcomes is limited and inconsistent, partly due to confounding factors. Some studies suggest that oxytocin‐induced labor increases pelvic floor muscle electrical activity during contractions, potentially contributing to obstetric injury.^15^ In our study, labor induction was significantly associated with urinary incontinence (OR: 3.07), consistent with findings from a prospective cohort of nulliparous women reporting an increased risk of pelvic floor disorders (OR: 1.5–2.3).^16^ However, other large prospective and randomized studies, including the ARRIVE trial follow‐up, found no significant association between oxytocin use during labor and postpartum pelvic floor symptoms or vaginal support.^17^, ^18^

Our study demonstrated an increased risk of UI in patients with postpartum urinary retention (OR: 4.04). This condition can result from periurethral and vulvar edema, as well as physiological changes during pregnancy that affect urethral pressure and length. Although no standardized guidelines exist, management typically begins with conservative methods, escalating to catheterization, if necessary, which increases the risk of infection.^19^

Implementing the perineal card as a routine screening tool at hospital discharge could improve the allocation of postpartum care by enabling early identification of women at higher risk of pelvic floor symptoms. This risk‐based approach may support targeted referral to pelvic floor rehabilitation while avoiding unnecessary interventions in low‐risk women, potentially reducing long‐term healthcare costs related to delayed diagnosis and chronic conditions. Although cost‐effectiveness was not evaluated, the tool's simplicity suggests good feasibility for routine use. Future studies should assess its economic impact and effects on healthcare utilization.

The robustness of this study is attributed to its prospective cohort design and substantial sample size. Additionally, the implementation of the perineal card at the time of discharge facilitated the prompt identification of symptomatic women and those possessing multiple risk factors, thereby enhancing their awareness and vigilance regarding potential PFDs.

This study had several limitations, including a selectively defined population and a sample size insufficient to develop a predictive model beyond the existing perineal card. Additionally, only a limited number of third‐ and fourth‐degree perineal lacerations were observed. In addition, obese women and neonates with a birth weight greater than 4000 g were excluded to ensure population homogeneity for this initial validation, which may limit the generalizability of the findings. Additionally, differences in follow‐up duration between symptomatic and asymptomatic women may have affected outcome detection. Given the encouraging results, future studies with larger and more diverse populations and standardized follow‐up intervals will be needed to confirm these findings and enhance generalizability.

The present study demonstrated that relying solely on traditional risk factors for postpartum pelvic floor dysfunction—such as obstetric anal sphincter injuries or urogynecologic symptoms at discharge—may lead to an underestimation of patients who would benefit from early evaluation and initiation of PFMT.

## 5 CONCLUSION

Pregnancy, particularly primiparity, is widely acknowledged as a significant risk factor for the development of pelvic floor dysfunction. This study evaluated the systematic implementation of the perineal card before discharge in a substantial cohort of women, emphasizing that current clinical practices frequently underestimate the number of patients at risk.

In light of these findings, our forthcoming objective is to design a multicenter validation study of the perineal card, incorporating a larger and more diverse sample, including multiparous women.

## AUTHOR CONTRIBUTIONS

M.S. and C.D. had the original idea for the study. M.A., L.D. and G.V. designed the study. M.C., M.S., F.B., S.F., D.V. and V.T. collected the data. M.I. and M.D.M. performed the analyses. M.A., D.A. and E.B. drafted the article which was then critically revised by L.D, G.V. and S.R. All authors approved the final version of the manuscript for publication.

## FUNDING INFORMATION

No external funding was received for this research.

## CONFLICT OF INTEREST STATEMENT

The authors declare no conflicts of interest.

## Supporting information


**Figure S1.** Italian Society of Urodynamics' (SIUD) delivery & pelvic dysfunction card – ENG version.
**Figure S2.** Italian Society of Urodynamics' (SIUD) Postpartum Screening Card – ENG version.
**Table S1.** Perineal card variables in study population.

## Data Availability

Research data are not shared.
