# Supplementary material for: An external validation study of a practical scoring system for identifying patients at risk of pelvic floor dysfunction following childbirth
Source: Int J Gynaecol Obstet. 2026 May 25;175(2):807–16. doi: 10.1002/ijgo.71099 (PMC13674847; doi:10.1002/ijgo.71099)
Supplement: Supplementary file 1 [file IJGO-175-807-s001.docx]

**An external validation study of a practical scoring system for identifying patients at risk of pelvic floor dysfunction following childbirth.**

Supplementary Materials – ENG version

Figure S1.. Italian Society of Urodynamics’ (SIUD) delivery & pelvic dysfunction card – ENG version

Figure S2. Italian Society of Urodynamics’ (SIUD) Postpartum Screening Card – ENG version

**SUMMARY TABLE**

Supplementary Table 1. Perineal card variables in study population.

| PERINEAL CARD | N (%) |
| --- | --- |
| GENERAL RISK FACTORS | 265 |
| Recurrent bronchitis / cough | 16 (6.0%) |
| Heavy physical labor | 53 (20%) |
| High-impact sports / intense physical activity | 43 (16.2%) |
| UROGYNECOLOGICAL RISK FACTORS |  |
| Urinary incontinence before pregnancy | 0 |
| Urinary incontinence in 1st/2nd trimester | 26 (9.8%) |
| Urinary incontinence in puerperium | 39 (14.7%) |
| Severe constipation before pregnancy | 37 (14%) |
| Severe constipation during pregnancy | 83 (31.3%) |
| Gas or stool incontinence | 5 (1.9%) |
| Recurrent urogenital infections | 26 (9.8%) |
| OBSTETRIC RISK FACTORS |  |
| Nulliparous | 265 (100%) |
| Weight gain >14kg | 98 (37%) |
| Breech/abnormal presentation | 0 |
| Sacral rotation | 13 (4.9%) |
| Precipitous/induced delivery | 142 (53.6%) |
| Analgesia | 192 (72.4%) |
| Second stage of labor > 60 min | 149 (56.2%) |
| Shoulder dystocia manoeuvres | 3 (1.1%) |
| Vacuum-assisted delivery | 54 (20.4%) |
| Kristeller manoeuvres | 31 (11.7%) |
| Medio-lateral episiotomy | 94 (35.5%) |
| Midline episiotomy | 0 |
| Intact perineum | 28 (10.6%) |
| Perineal laceration – Grade 1 | 67 (25.3%) |
| Perineal laceration – Grade 2 | 68 (25.7%) |
| Perineal laceration – Grade 3 | 9 (3.4%) |
| Perineal laceration – Grade 4 | 1 (0.4%) |
| Perineal hematoma | 1 (0.4%) |
| Perineal infection | 1 (0.4%) |
| Catheterization | 126 (47.5%) |
| Manual removal of a retained placenta | 6 (2.3%) |
| Neonatal head circumference >35 | 53 (20.0%) |
| Neonatal weight > 4000 gr | 0 |
| Perineal card score (median(range)) | 10 (2-25) |
